# Delays in switching patients onto second-line antiretroviral treatment at a public hospital in eThekwini, KwaZulu-Natal

**DOI:** 10.4102/sajhivmed.v18i1.696

**Published:** 2017-03-31

**Authors:** Denver Narainsamy, Saajida Mahomed

**Affiliations:** 1 School of Nursing and Public Health, University of KwaZulu-Natal, South Africa; 2 School of Laboratory Medicine and Medical Sciences, University of KwaZulu-Natal, South Africa

## Abstract

**Background:**

South Africa has one of the largest antiretroviral treatment (ART) programmes globally. In addition to increasing access to ART, it is important that the health system also focuses on the appropriate management of patients who fail first-line ART. Delays in switching patients onto second-line ART can adversely affect patient outcomes.

**Aim:**

To identify the patient-related and programmatic factors that delay switching patients onto second-line ART, and to assess whether these delays contribute to subsequent virological failure.

**Methods:**

Clinical records of adult patients switched onto second-line ART between 2011 and 2014 at a public antiretroviral clinic were used to collect demographic, clinical, laboratory and programmatic data (availability of viral load results, inadequate patient follow-up, insufficient notes for effective follow-up). Data were analysed using univariate and multivariate logistic regression.

**Results:**

The median duration from the date of first and confirmatory documented high viral load (VL > 1000 copies/mL) to being switched to second-line ART was 13.2 months [interquartile range (IQR) 1.1–52.7 months] and 6.4 months (IQR 0–43.3 months), respectively. Inadequate prescriber notes for appropriate follow-up (*p* = 0.01) and unavailability of patients’ viral load results (*p* = 0.02) were significantly associated with delays in switching to second-line ART. There was no significant association between the time taken to switch to second-line ART and subsequent virological failure.

**Conclusion:**

We observed lengthy delays in switching patients to second-line ART. Modifiable programmatic factors were found to be significantly associated with delays in switching to second-line ART.

## Introduction

In 2011, it was estimated that more than eight million people were accessing antiretroviral treatment (ART) in low- and middle-income countries.^1^ This rapid scale-up of ART initiation at a global level has significantly improved health outcomes for the majority of those living with human immunodeficiency virus (HIV) and acquired immunodeficiency syndrome (AIDS). However, the critical public health challenge must now shift to managing the increasing number of patients that are likely to fail first-line ART.^2^

South Africa has one of the largest ART programmes globally with more than three million people currently accessing treatment. In addition to having the largest HIV epidemic worldwide, with an estimated 6.2 million people infected,^3^ South Africa has a large burden of communicable diseases, non-communicable diseases, violence and injury, and perinatal and maternal diseases.^4^ These challenges have conveyed significant pressures on the delivery of healthcare within the country.

The current National ART Guidelines advocate an early switch to second-line ART in the face of virological failure. Two consecutive viral loads (VLs), taken two months apart, that are greater than 1000 copies/mL serve as confirmation of virological failure, provided that factors related to adherence, drug toxicities and drug interactions are adequately addressed.^5^ Ideally, an adult patient should be switched to second-line ART within a period of three months from the date of first documented high VL. However, limited research has demonstrated delays in switching patients to second-line ART.^2,6^

Although theoretical principles suggest that the delay in switching patients to second-line ART can result in the accumulation of resistant mutations that may compromise second-line ART,^7^ this is unlikely in the context of the South African ART programme as cross-resistance between first-line and second-line drugs are unlikely.^8^ However, limited research from South Africa and other African countries have shown that patients maintained on a failing first-line regimen for long periods had an increased risk of mortality after switching to a protease inhibitor (PI)-based second-line regimen.^9,10^ These findings highlight the importance of timely switching of patients who are failing on first-line ART to second-line ART. We investigated the programmatic and patient-related factors that contribute to delays in switching patients to second-line ART and assessed the association between these delays and subsequent virological suppression.

## Methods

### Study setting and population

The study was conducted at an outpatient antiretroviral clinic situated in a public hospital in the eThekwini Health District, KwaZulu-Natal (KZN), South Africa. The area consists of a peri-urban setting with more than 6500 patients accessing ART services at this hospital in 2014.

Adult patients (≥ 18 years) attending the clinic who were on a non-nucleoside reverse transcriptase inhibitor (NNRTI)–based first-line ART regimen and switched onto a PI-based second-line ART regimen between January 2011 and January 2014 as a result of virological failure were included in the study. These patients were identified using pharmacy records,^1^ electronic databases^2^ and active patient identification during prescription refills. Patients who had been referred from primary healthcare clinics owing to virological failure were excluded from the study as their medical records were not complete.

### Study design and data collection

A retrospective cohort study design was used. Patient’s medical records were accessed to obtain demographic, clinical and programmatic data. The data were collected with the assistance of a trained research nurse using a standardised data collection tool. Clinical variables recorded included baseline CD4+ count, baseline VL, first-line ART regimen, regimen modifications prior to switch to second-line ART (changes made to the standard first-line ART regimen owing to adverse drug reactions or drug interactions), date of first and second documented high VL, date of switch to second-line ART, VL at switch to second-line ART and VL at least 12 months after switch to second-line ART. Pregnancy and tuberculosis co-infection were also recorded. Patient-related variables included age, gender and patients defaulting their appointment (defined as patient failing to attend the clinic within 30 days of their set appointment date during the period of first documented high VL and switch to second-line ART). The programmatic variables recorded included the availability of VL results, inadequate prescriber notes for appropriate follow-up (defined as the attending clinician failing to document a management plan in the patient’s medical record from the date of first documented high VL, by either not referring the patient for step-up adherence counselling, not documenting the need for a repeat VL after two months and not documenting the high VL in the patient’s medical record), inadequate prescriber follow-up at the subsequent visit (defined as the attending clinician failing to follow the patient’s clinical management plan of the previous visit by either not repeating a VL test or not switching second-line ART after reviewing the repeated VL result) and the patient’s medical record being mislaid. Time from first and second documented high VLs to time of switch to second-line ART was measured and the VL measurement at least one year after switch to second-line ART was recorded.

### Statistical analysis

The association between delays in switching to second-line ART and subsequent virological failure was determined using incidence risk ratios with 95% confidence limits and inferential statistical analysis using Pearson’s chi-square tests. Multivariate analysis (Poisson regression) was performed to determine if any of the patients or programmatic variables was independently associated with the development of virological failure. All statistical analyses were performed using STATA version 13 (Stata Corporation, College Station, Texas, USA).

## Ethical considerations

The research was approved by the Biomedical Research and Ethics Committee of the University of KwaZulu-Natal (BE 337/14). Gatekeeper permission was obtained from the hospital’s Chief Executive Officer and the KwaZulu-Natal Provincial Health Research and Ethics committee.

## Results

**FIGURE 1:**
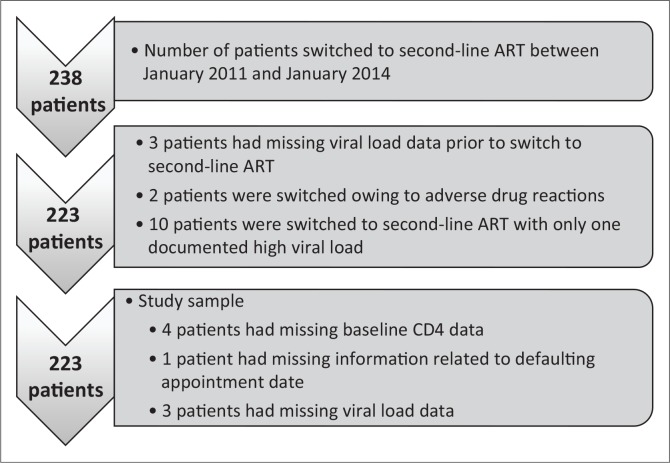
Summary of study sample.

A total of 238 patients were switched to second-line ART during the three-year period. Fifteen patients were excluded because of missing data (Figure 1). Of the remaining 223 patients, 68% (*n* = 152) were female (Table 1). The median age for males and females was 42 [interquartile range (IQR) 19–73 years] and 36 years (IQR 18–57), respectively (*p* < 0.01). The median baseline CD4 count of patients was 94 cells/mm^3^ (IQR 2–384).

**TABLE 1:** Characteristics of patients switched to second-line antiretroviral treatment at an antiretroviral clinic in eThekwini Health District, 2011 to 2014.

Patient characteristic	Variable	*n*	(%)
Gender	Male	71	31.9
	Female	152	68.1
Age (years)	18–29	35	15.7
	30–39	103	46.2
	40–49	66	29.6
	> 49	19	8.5
Baseline CD4 count (cells/mm^3^)	0–100	131	59.8
	101–200	65	29.7
	> 200	23	10.5
History of TB	Yes	73	33.2
Pregnant (during period of virological failure)	Yes	21	14
First-line ART exposure ever	3TC-TDF-EFV	68	30.5
	3TC-TDF-NVP	43	19.3
	3TC-AZT-EFV	7	3.1
	3TC-AZT-NVP	8	3.6
	3TC-D4T-EFV	67	26
	3TC-D4T-NVP	58	26
	3TC-ABC-EFV	1	0.4
First-line ART exposure at switch	3TC-TDF-EFV	68	30.5
	3TC-TDF-NVP	40	17.9
	3TC-AZT-EFV	6	2.7
	3TC-AZT-NVP	8	3.5
	3TC-D4T-EFV	55	24.6
	3TC-D4T-NVP	45	20.2
	3TC-ABC-EFV	1	0.4
Regimen modification prior to switch	Yes	29	13
Patient defaulting their appointment	Yes	38	17.1
Blood results available (VL)	Yes	196	87.9
Inadequate prescriber notes for effective follow-up	Yes	126	56.5
Inadequate prescriber follow-up at subsequent visit	Yes	166	74.4
Patients medical records lost or misplaced	Yes	9	4
Viral load at switch (copies/mL)	< 10 000	45	20.2
	10 000–100 000	117	52.5
	> 100 000	61	27.3

TB, tuberculosis; ART, antiretroviral treatment; VL, viral load; TDF, tenofovir; EFV, efavirenz.

### Delays in switching to second-line antiretroviral treatment

The median duration from the date of first documented high VL and switch to second-line ART was 13.2 months (IQR 1.1–52.7 months), and the median duration from the time of confirmed virological failure (second documented high VL) and switch to second-line ART was 6.4 months (IQR 0–43.3 months). The median time interval between first and second documented high VL was 6.8 months (IQR 0.1–32 months). Eighty patients (36%) experienced delays of greater than six months from the date of confirmed virological failure to the date of switch to second-line ART.

In bivariate analysis, there were no significant associations between the clinical or patient-related factors and delays in switching to second-line ART. Patients whose medical records contained insufficient prescriber notes for appropriate follow-up demonstrated a 1.8-fold higher risk of experiencing delays in switching to second-line ART (95% CI, 1.21–2.68, *p* = 0.004) when compared to patients whose medical records had sufficient prescriber notes. In multivariate analysis, both insufficient prescriber notes for appropriate follow-up (RR 1.94, 95% CI, 1.16–3.24, *p* = 0.01) and non-availability of VL results (RR 1.66, 95% CI, 1.08–2.56, *p* = 0.02) were significantly associated with delays in switching to second-line ART (Table 2).

**TABLE 2:** Patient-related and programmatic variables associated with delays in switching patients to second-line antiretroviral treatment at an antiretroviral clinic in eThekwini Health District, 2011 to 2014.

Factor	RR (95% CI)	*p*-value	Adjusted RR (95% CI)	*p*-value
Gender (females)	1.2 (0.8–1.9)	0.29	0.2 (0.002–27)	0.33
**Age range (years)**				
18–29	1.1 (0.5–2.4)	0.84	0.9 (0.4–2.0)	0.80
30–39	1.3 (0.6–2.6)	0.48	1.1 (0.6–2.3)	0.73
40–49	0.9 (0.4–2.0)	0.92	0.8 (0.4 –1.6)	0.47
Patient defaulted appointment	1.3 (0.9–2.0)	0.19	1.4 (0.9 –2.5)	0.21
Blood results unavailable	1.4 (0.9 –2.2)	0.13	1.7 (1.1 –2.6)	0.02
Insufficient notes for follow-up	1.8 (1.2 –2.7)	0.004	1.9 (1.2 –3.2)	0.01
Inadequate patient follow-up	1.4 (0.9 –2.2)	0.18	1.1 (0.6 –1.9)	0.79
Patients records lost	1.3 (0.6 –2.7)	0.56	1.2 (0.5 –2.7)	0.70

### Virological failure

**FIGURE 2:**
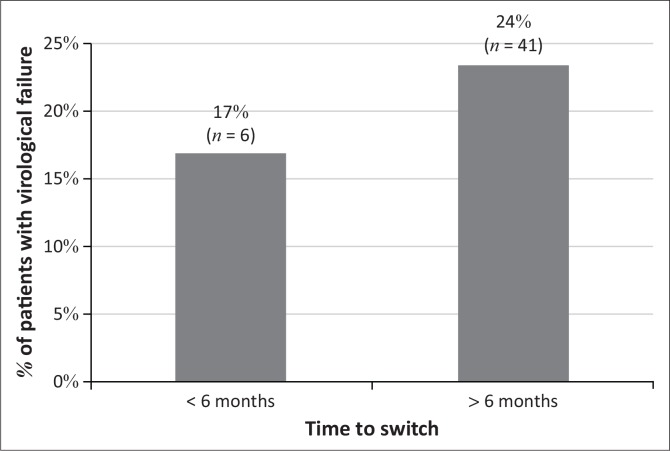
Virological failure among patients switched to second-line antiretroviral treatment at an antiretroviral clinic in eThekwini health district, 2011 to 2014.

Of the 223 patients in the cohort, almost a quarter (21%, *n* = 47) developed virological failure within 18 months of being switched to second-line ART. The majority of these patients (87%, *n* = 41) had more than a six-month delay to switching to second-line ART from the date of first documented high VL. (Figure 2). Patients with delays greater than six months demonstrated a 1.6-fold higher risk of developing virological failure when compared to patients switched within six months (95% CI, 0.71–3.54, *p* = 0.26). There was no significant linear correlation between the time taken to switch to second-line ART and virological failure (*r* = 0.027). In bivariate analysis, patients that defaulted their appointment dates demonstrated a 1.9-fold higher risk of virological failure compared to patients that did not default their appointment dates (95% CI, 1.10–3.21, *p* = 0.02). However, in multivariate analysis, this association was not significant *(p* = 0.11) (Table 3).

**TABLE 3:** Programmatic, patient-related and clinical variables associated with virological failure at an antiretroviral clinic in eThekwini Health District, 2011 to 2014.

Factor	RR (95% CI)	*p*	Adjusted RR (95% CI)	*p*
**Gender (females)**	**0.8 (0.5–1.4)**	**0.47**	**0.08 (< 0.01–404)**	**0.56**
**Age range (years)**	**-**	**-**	**-**	**-**
18–29	1.5 (0.6–4.1)	0.37	1.9 (0.6–6.2)	0.24
30–39	0.8 (0.3–2.1)	0.64	0.9 (0.3–2.7)	0.87
40–49	0.9 (0.3–2.4)	0.86	0.9 (0.3–2.7)	0.85
**Viral load at switch**	**1.1 (0.9–1.3)**	**0.45**	**1.1 (0.8–1.7)**	**0.53**
History of TB	0.9 (0.6–1.6)	0.84	0.8 (0.5–1.4)	0.40
Pregnancy	1.0 (0.9–1.1)	0.49	0.9 (0.3–2.9)	0.92
Regimen modification prior to switch	0.9 (0.5–2.1)	0.93	0.9 (0.4–2.0)	0.83
**First-line regimen**	**-**	**-**	**-**	**-**
Exposure to EFV-based first-line regimen	1.3 (0.8–2.3)	0.29	1.4 (0.8–2.5)	0.28
Delays of greater than six months	1.7 (0.8–3.8)	0.18	1.6 (0.7–3.5)	0.26
Patients defaulted appointment	1.9 (1.1–3.2)	0.02	1.5 (0.9–2.7)	0.11

TB, tuberculosis; EFV, efavirenz.

Patients in the age group 18–29 years had a 1.9-fold higher risk of developing virological failure when compared to patients in the oldest age category (95% CI, 0.63–6.20, *p* = 0.24).

## Discussion

Our study is one of the few local studies that investigated programmatic and patient-related factors that could delay switching patients to second-line ART. The median time taken to switch patients onto second-line ART (6.4 months from the date of confirmed documented high VL) is greater than the reported median time in other studies conducted within South Africa. In Khayelistha, Cape Town, the average time taken to switch patients to second-line ART was 5.3 months (IQR 2.2–11.2).^6^ A large, multi-centre study conducted across five sites in Durban, Cape Town and Gauteng reported an average time of 4.6 months (IQR 2.1–8.7) before patients were switched to second-line ART.^2^ The median time between the first and second documented high viral was 6.8 months which is longer than the two months time advocated in the National ART Guidelines.^5^

We identified significant associations between modifiable programmatic factors and the delay in switching patients to second-line ART. The unavailability of VL results delays clinical decision-making and can potentially compromise patient care. This finding highlights the need for information technology and telecommunication networks to support healthcare delivery within ART clinics in KZN.

Insufficient prescriber notes is a concern, especially as patients often do not see the same healthcare provider on their follow-up visit. The poor documentation of a comprehensive patient treatment plan or inadequate patient follow-up in the face of an available treatment plan is an avoidable occurrence that can compromise patient care. These factors, together with the delays in confirming virological failure and switching patients to second-line ART, highlight the absence of robust systems necessary to manage chronic diseases like HIV within the public healthcare setting in South Africa. Studies within Africa have demonstrated that only 2.4% of patients are switched to second-line ART,^11^ in contrast to the World Health Organization’s estimates that 9.4% of patients are likely to fail first-line ART after 12 months on treatment.^12^

The South African ART programme does not allow for genotypic testing at first-line ART failure; this therefore limits our interpretation of the virological failure seen in 21% of the cohort after switching to second-line ART. We observed no significant association between delays in switching patients to second-line ART and subsequent virological failure. Although studies demonstrate that maintaining a patient on a failing first-line regimen for prolonged periods may lead to the accumulation of complex mutations that would compromise the nucleoside reverse transcriptase inhibitor (NRTI) backbone of second-line ART,^8,13^ this appears to have less impact in South Africa. This finding is consistent with the current literature which demonstrates that the majority of resistance patterns seen at first-line ART failure does not confer resistance to South Africa’s second-line ART drugs.^8^ In addition, studies have documented very low rates of drug resistance to a protease-based second-line regimen.^14^

We observed that patients who defaulted their appointment dates (a surrogate marker for adherence) demonstrated a higher risk of virological failure when compared to patients that did not. Although this increased risk was not significant, when combined with the short follow-up period within the study (less than 18 months after switch to second-line ART), the most plausible reason for second-line virological failure points to issues of adherence rather than drug resistance.

Although the increased risk of virological failure amongst younger patients was not statistically significant, this finding supports the available body of evidence which suggests that patients in younger age groups demonstrate poor adherence to ART when compared to older patients, subsequently making them more susceptible to drug resistance and virological failure.^15^

The study limitations were related to the retrospective nature of the study design. Patients with virological failure but not switched onto second-line ART owing to being transferred to another healthcare facility, defaulting or death were excluded. The use of data of patients defaulting their appointment dates is not an accurate reflection of adherence to treatment. We assumed that virological failure to second-line ART was acquired as it was not possible to determine if a patient was infected with a resistant strain of HIV. However, it is unlikely that transmitted resistance would have affected the study results given the low rates of transmitted resistance to NNRTIs, with little or no transmitted resistance documented for PIs.^16,17^ To assess the delays in switching patients to second-line ART, we looked at the clinician’s documentation of a high VL and the availability of VL results in patients’ medical records. It is possible that a patient’s VL result may have been available but not documented or filed in the patient’s medical record. This may have either overestimated or underestimated the time taken to switch to second-line treatment. We were unable to analyse patients’ baseline VL results in relation to virological failure as changes to the South African ART guidelines in 2012 eliminated the need for baseline VLs when initiating patients onto ART.^5^ Although the study was limited to one antiretroviral clinic in eThekwini Health District, it is likely to represent many other antiretroviral clinics in the district as most of the clinics face similar challenges. In more rural settings, the delay in switching patients to second-line ART is likely to be longer as a result of the reduced availability of skilled human resources^18,19^ and the absence of telecommunication networks to support clinical decision-making.

## Conclusion and recommendations

Our study demonstrated considerable delays in switching patients to second-line ART. The programmatic factors associated with these delays are preventable. Strengthening documentation and record keeping through the training of healthcare professionals remains essential in addressing the gaps associated with chronic disease management, in addition to alleviating the medico-legal implications of such practices. Upgrading and integrating of the information technology infrastructure within the public healthcare sector will allow for a more confident and responsive healthcare system that will improve the clinical management of patients.

Further investigation into the long-term outcomes of patients with delayed switching to second-line ART is warranted, with emphasis on more accurate methods of assessing patient adherence.
